# Workplace Health Promotion Programs Targeting Smoking, Nutrition, Physical Activity, and Obesity in Men: A Systematic Review and Meta-Analysis of Randomized Controlled Trials

**DOI:** 10.1177/10901981231208396

**Published:** 2023-11-27

**Authors:** Aaron Bezzina, Erin D. Clarke, Lee Ashton, Trent Watson, Carole L. James

**Affiliations:** 1 Centre for Resources Health and Safety, College of Health, Medicine and Wellbeing, University of Newcastle, Callaghan, 2308, Australia; 2 School of Health Sciences, College of Health, Medicine and Wellbeing, University of Newcastle, Callaghan, 2308, Australia; 3 Food and Nutrition Program, HMRI, Lot 1 Kookaburra, New Lambton Heights, NSW 2305, Australia; 4 Active Living Research Program, Hunter Medical Research Institute (HMRI), Lot 1 Kookaburra Circuit, New Lambton Heights, NSW 2305, Australia; 5 School of Education, College of Human and Social Futures, University of Newcastle, Callaghan, 2308, Australia; 6 Ethos Health, Newcastle West, 2302, Australia; 7 Sydney School of Health Sciences, Faculty of Medicine and Health, The University of Sydney, Camperdown NSW, 2006, Australia

**Keywords:** workplace, men, review, smoking, nutrition, physical activity, alcohol, obesity

## Abstract

The workplace has been highlighted as a potential setting to deliver health promotion programs to target modifiable health behaviors that contribute to chronic disease. This review evaluated the effectiveness of interventions implemented within the workplace that targeted either smoking, nutrition, alcohol, physical activity, and/or overweight and obesity in men. A review protocol was prospectively registered through PROSPERO (CRD42021293398). Five electronic bibliographic databases were searched for randomized controlled trials conducted in the workplace assessing chronic disease risk factors in men from January 2010 to August 2021. Eleven studies were included, reporting on overweight and obesity *n* = 8, physical activity *n* = 7, nutrition *n* = 4, alcohol *n* = 3, smoking *n* = 3, with eight studies assessing multiple outcomes. Results were mixed. Narrative synthesis highlighted studies reporting improvements to snacking frequency, sugar sweetened beverage consumption, and physical activity (METs and Vo2 max). Meta-analysis highlighted pooled mean decrease in body weight of −0.28 kg up to 3 months; −1.38 kg for >3 months, and pooled mean decrease in body mass index 0.06 kg/m^2^ up to 3 months; −0.27 kg/m^2^ for >3 months. Despite the encouraging direction of the relationship, results were not statistically significant (*p* > .05). Findings underscore the potential of workplace health promotion programs targeting certain chronic disease risk factors in men; however, future research should consider long-term study designs to assess the efficacy of workplace health programs as a solution to the growing burden of global disease.

## Introduction

Global figures continue to report the pervasive nature of noncommunicable diseases (NCDs), accounting for an estimated 71% of all deaths globally (World Health Organization [WHO], 2022). The modifiable nature of many NCDs means prevention programs targeting smoking cessation, unhealthy diets, alcohol use, physical inactivity and, overweight and obesity can decrease lifestyle related risk factors (GBD 2017 Disease and Injury Incidence and Prevalence Collaborators, 2018). In recent years, there has been an increased public health focus on the management of NCDs (WHO, 2022). This includes workplaces being identified as a priority setting for the delivery of health promotion initiatives (Peñalvo et al., 2021).

Workplace health promotion (WHP) programs refer to a set of strategies and programs that look to meet and build upon the health and safety needs of employees throughout their employment (Centers for Disease Control and Prevention, 2016). Workplaces offer the necessary infrastructure and sustained reach to a large number of individuals for prolonged periods each day (WHO, 2020). This larger reach also means workplaces’ have access to demographics who would otherwise be difficult to engage in health promotion. Blue-collar male dominated industries such as mining (Bezzina et al., 2021), construction (Viester et al., 2018), and manufacturing (Morgan et al., 2011) could all potentially benefit from WHP programs as this population is usually underrepresented in health research (Van der Put et al., 2020), yet may benefit the most from such interventions (Shen et al., 2018).

Men are heavily underrepresented in health research, with reviews suggesting that males only account for 20% of health behavior research (Maher et al., 2014; Ryan et al., 2019). This has far reaching implications when it comes to designing WHP programs that are tailored to men’s specific health needs and may further compound health inequalities between sexes. This is further underpinned by the 2018 “Health and well-being of men in the WHO European Region: better health through a gender approach” report (WHO, 2018), which reinforces that biology alone cannot explain the health inequalities experienced between men and women. Regardless of sex, health outcomes for both men and women are implicated by a multitude of elements including behaviors, access to care, country, and social networks (WHO, 2018). Research that is focused solely on males is a crucial step in addressing these inequalities.

Despite the reported potential of WHP programs in men (Bezzina et al., 2022; Morgan et al., 2011; Viester et al., 2018), there have been no systematic review or meta-analysis conducted to assess the pooled effects of these programs. Workplace health promotion programs are primed to bridge the gap between engaging men in health promotion and lowering lifestyle-related risk factors. Considering the novel approaches to health promotion that workplaces can necessitate, and the differences in how men and women respond to health, the aims of the current review were to:

1. Evaluate the effectiveness of WHP programs targeting smoking, nutrition, alcohol, physical activity, and/or overweight and obesity risk factors in men.
2. Identify patterns regarding intervention design and effectiveness of programs.

## Methods

### Search Strategy and Selection Criteria

The methods undertaken followed the PRISMA (Preferred Reporting Items for Systematic Reviews and Meta-Analyses) guidelines, and the protocol was registered with PROSPERO (CRD42021293398).

To be included in this review, articles had to examine the effectiveness of wellness interventions undertaken in the workplace. Workplaces could be from any employment sector: manufacturing, health, education, business, information technology, retail, agriculture, construction, or mining. Studies conducted within a military setting were excluded due to the highly controlled nature of the military activities, and the lack of generalizability to other workplace environments. Studies had to be conducted within an Organization for Economic Co-operation and Development (OECD) country focusing strictly on all male populations. Studies which stratified results by sex were excluded. Articles focused on population groups selected based on pre-existing co-morbidities were excluded (i.e., type 2 diabetes only).

Articles which examined the impact of a health promotion interventions, specifically (a) smoking cessation, (b) nutrition, (c) alcohol, (d) physical activity, and (e) overweight and obesity, were included. Examples of interventions include those that target the structures or infrastructures within worksites (e.g., changes to stairways), or the socio-cultural aspect of the workforce-wide social norms or communications (e.g., policies on active/green commuting to work). Studies undertaken in laboratory or highly controlled environments were excluded. Comparison groups with no intervention (e.g., waitlist control) and/or active treatments were considered for inclusion.

Outcomes of interest were related to measurement and assessment of risk factors associated with lifestyle diseases. This included diet (fruit and vegetables, sugar sweetened beverages/diet drinks, energy-dense nutrient-poor foods, energy (calories), fiber, physical activity (minutes active [moderate, vigorous] resistance training, MET minutes), alcohol consumption, tobacco smoking and weight/adiposity (weight, body mass index [BMI], waist circumference [WC], body fat [BF] percentage). Key subjective and objective assessments of the outcomes were included. Measures of behavioral intention, preferences, knowledge, and attitudes toward a health risk behavior were excluded. This review looked exclusively at randomized controlled trials. All other nonrandomized experimental study designs without a control or comparator were excluded.

Five electronic bibliographic databases were searched including Medline; Embase; Cochrane library, Scopus and CINAHL in August 2021. The search strategy (Supplementary Materials 1) was developed by the research team with consultation from a medical librarian and included four arms: (a) male terms, (b) workplace terms, (c) intervention terms, and (d) exposure terms. Several limitations were imposed on the search including peer-reviewed journal articles in English as well as publication from January 2010 and beyond. The decision to include studies from 2010 onwards was based upon frameworks and models released by the World Health Organization which impacted design and delivery of WHP programs from 2010 onwards through a coordinated and systematic approach to workplace wellness (Burton, 2010). This is in addition to the growing amount of contemporary literature being published in this space as evidenced by Peñalvo et al. (2021), which in their review of 121 studies, highlights that 60% (*n* = 72) of included studies being published since 2010, with lesser numbers published between 2000 and 2009 (*n* = 31, 26%) and before 2000 (*n* = 18, 15%).

The research team consulted with the medical librarian during the entire process in testing different keywords, MeSH, and filter terms, to achieve the most sensitive and specific search strategy. A search of reference lists of included papers and relevant systematic reviews was also undertaken. Retrieved articles for each database were exported to endnote and duplicates were eliminated.

Two independent reviewers (A.B. and E.C.) assessed the title, abstract, and keywords of all identified papers. Full text articles were retrieved for records that appeared relevant or unclear. Two reviewers independently assessed these for inclusion/ exclusion (A.B. and E.C.), with reasons for exclusion recorded. Conflicts were resolved via consensus between reviewing authors. Where insufficient detail to allow determination of eligibility was provided, the corresponding author was contacted to confirm (*n* = 6), with papers excluded from nonresponding authors.

The Cochrane Collaboration RoB 2 tool was completed by two independent reviewers (A.B. and E.C.) (Sterne et al., 2019), with differences resolved via consensus. Risks of bias results are presented by individual risk components across all studies (as low risk, some concerns, or high risk of bias), and were grouped based upon whether studies employed an intention to treat strategy or per protocol.

### Data Extraction and Meta-Analysis

One reviewer (E.C.) extracted data, which were cross-checked by a second reviewer (A.B). Data extraction included study characteristics (country, industry, length of study, length of follow-up, retention percentage), participant data, study design, intervention components, theoretical frameworks, and outcomes.

To be included in the meta-analysis, studies needed to report mean and standard deviation at all timepoints. Six studies reported their results as mean change only; hence the authors contacted the corresponding author of these studies to ascertain these specific measures (mean and standard deviation at all timepoints), with only three responding. Considering the novel area of research endeavored by this review, and the already limited scope of literature in this area, this method was chosen to help bolster the number of measures in the meta-analysis, and to make results more robust and generalizable. For all outcomes aside from weight and BMI, there were insufficient comparable studies for a meta-analysis. Therefore, results are described in narrative form for included studies.

Meta-analysis to assess change in bodyweight (kg) and BMI (kg/m^2^) was conducted for both intervention and comparator/control groups at each time point. The meta-analysis was conducted using R statistical software (V 4.1.2, Vienna, Austria) using the Metafor package (V 2.0, Vienna, Austria). For each study, the effect at baseline and each time point, expressed as months post baseline, was estimated as the mean difference (mean intervention group-mean control group) using the unbiased option for variance estimates. To account for multiple measures per study, a series of multilevel models were investigated with nested random effects top level being study, then treatment type and time period using restricted maximum likelihood (REML) estimation. The final model contained study and time as nested random effects as the treatment type random effect had zero variance. Moderator variables evaluated as fixed effects in the model were treatment type (nutrition education, multicomponent), and time was treated as a categorical variable in two versions (as originally reported with seven different timepoints for weight and six timepoints for BMI) and a simplified grouped form with baseline, up to 3 months and greater than 3 months, with interaction between these two tested. There was substantial correlation between time periods, with the time random effect similar in size to study random effects. Hence, the moderator effect for the three-group version of time was estimated at each of the two follow-up time periods as difference from baseline. Residual plots were used to examine homogeneity of variance and normality assumptions. To assess the presence of publication bias, funnel plots were created, visually inspected, and rank correlation was used to assess for funnel plot asymmetry. Heterogeneity between studies was observed from the forest plots.

## Results

**Figure 1.**
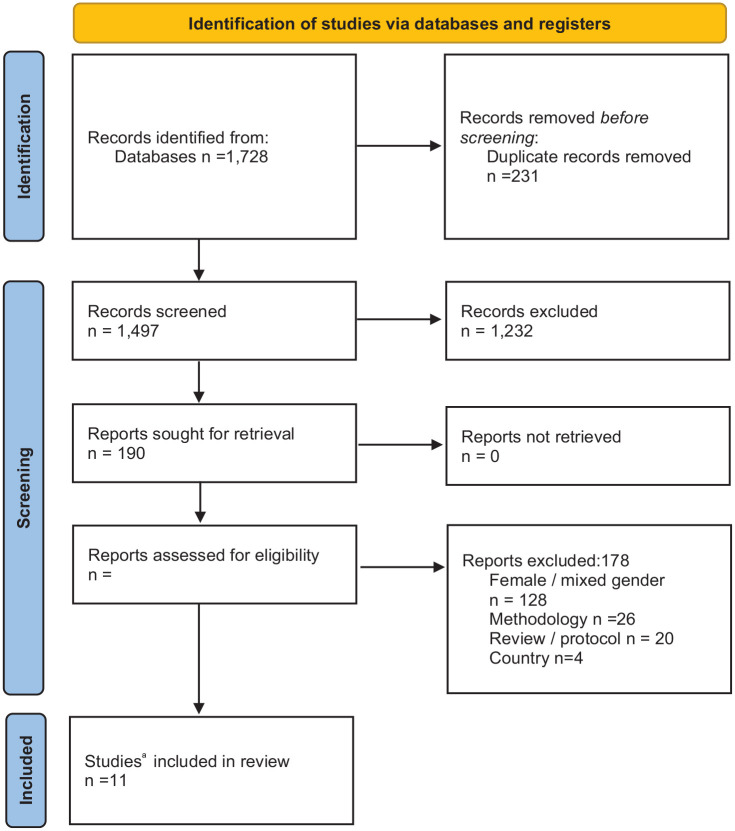
Preferred Reporting Items for Systematic Reviews and Meta-Analyses (PRISMA) Flow Diagram of Included Studies ^a^ Morgan et al. (2011) & Morgan et al. (2012) reported the same study outcomes across two different papers. Only Morgan et al. (2011) has been included in quantitative analysis.

The search identified 1,728 abstracts. After duplicates were removed, 1,497 records were screened with 1,232 abstracts excluded based on title /abstract, leaving 190 records. From this, 12 articles and 11 individual studies remained after full text screening (exclusion reasons outlined in Figure 1). Of the 11 included studies, six studies were eligible for meta-analysis of weight, and five were eligible for meta-analysis of BMI.

Across included studies, overweight/obesity outcomes were the most widely reported (*n* = 8), followed by physical activity (*n* = 7), nutrition (*n* = 4), alcohol (*n* = 3), and smoking (*n* = 2). Majority of studies were published between 2010 and 2014 (*n* = 5), followed equally by 2015 and 2019 (*n* = 3) and 2020 and 2021 (*n* = 3). Studies were conducted in several different countries including United States (*n* = 1), Australia (*n* = 1), Netherlands (*n* = 2), South Korea (*n* = 3), Japan (*n* = 2), Ireland (*n* = 1), and Denmark (*n* = 1). Most studies were conducted in a construction setting (*n* = 4) or an office setting (*n* = 4), followed by mixed working environments (*n* = 2) and manufacturing (*n* = 1).

Across the included studies, there were 1,932 participants (median: 101, range 22–816). Intervention duration was either 0 to 3 months (*n* = 5) or 3.25 to 6 months (*n* = 6). Only three studies employed a follow-up beyond the end of the intervention period, with the mean post intervention follow-up being 5 months in duration. The mean retention rate was 92% (range 60% to 100%) at intervention completion. Several theories were utilized during intervention design including Social Cognitive Theory (*n* = 4), Transtheoretical Model (*n* = 2), Socio-Ecological Theory (*n* = 1), and Intervention Mapping (*n* = 1). Studies focused on weight loss/maintenance (*n* = 4), followed closely by physical activity (*n* = 3), and wellness more broadly (*n* = 3). Only one study exclusively focused on smoking cessation, with no other studies solely concerned with nutrition or alcohol. Supplementary Materials 2 summarizes study characteristics grouped by risk factors.

There were five articles which assessed intention-to-treat results (Supplementary Materials 3 and 4). For randomization process, there was a low risk of bias, with more than half the studies (*n* = 3, 60%) adequately describing randomization processes and sequence concealment. When assessed for deviations from intended interventions 100% of studies (*n* = 5) reported low risk, indicating they followed the design set out in the trial protocol. In addition, 100% of studies scored low risk for missing outcome data and selection of reported results (*n* = 5). Furthermore, 100% (*n* = 5) of studies reported low risk of bias for measurement of the outcome, indicating differential measurement errors were low. Overall bias was low, with 60% (*n* = 3) ranking as low risk of bias and 40% (*n* = 2) having some concerns.

There were six articles which assessed per-protocol results (Supplementary Materials 5 and 6). For randomization process, there was a low risk of bias, with more than half the studies (*n* = 4, 66.7%) adequately describing randomization processes and sequence concealment. When assessed for deviations from the intended interventions, results were mixed, with half ranking as low risk (*n* = 3, 50%). Missing outcome data were sufficiently described with all but one study ranking as low risk (*n* = 5, 83.3%). Most studies in the per-protocol group were ranked as high risk (*n* = 4, 66.7%) regarding outcome measurement, which could be due in part to how data was collected, or appropriateness of measurement tools utilized. Most studies were low risk for the selection of the reported results (*n* = 5, 83.3%). Overall, more than half of studies in the per-protocol group were ranked as high risk of bias (*n* = 4%, 66.7%) with the remaining low risk (*n* = 2, 32.3%).

### Effectiveness on Outcomes

Seven studies reported a physical activity outcome (Gram et al., 2012; Groeneveld et al., 2011; Kim et al., 2015; Maruyama et al., 2010; Morgan et al., 2011; Nicolson et al., 2021; Viester et al., 2018); however, items reported varied among studies. Nearly half (*n* = 3, 43%) of these studies reported a statistically significant between-group difference favoring the intervention (Gram et al., 2012; Morgan et al., 2011; Viester et al., 2018). Gram et al. (2012) illustrated significant improvements to Vo2max. Morgan et al. (2011) reported significant improvements to METs and physical activity levels, while Viester et al. (2018) showed significant improvements to percentage of individuals meeting physical activity guidelines for vigorous activity. It should be noted that both Morgan et al. (2011) and Viester et al. (2018) used self-reported tools to measure physical activity outcomes.

**Table 1.** Summary of Study Characteristics and Findings.

First author (year) Country Setting	Primary outcome Intervention (I)/Comparator (C)	Study characteristics	Results
Smoking			
Asfar et al. (2020) United States Construction	Outcome: Self-reported prolonged abstinence (no smoking) I) (a) One face-to-face behavioral group counseling session, (b) two brief follow-up phone calls and were referred to tobacco quit line (QL) for standard QL intervention (up to four phone counseling sessions and free 2-week supply of NRT) (c) 6 weeks supply of NRT. C) (a) Standard care referral to QL, 2 free weeks of NRT, and participants (b) extra 6 weeks’ supply of NRT.	8 weeks *N =* 122 3 months 65% (*n =* 85), 6 months 72% (*n =* 96) 0, 3 m, 6 m	**ITT: Between-group abstinence rates at 3 months:** Intervention: 44%, control: 38%, *p =* .571. ITT: Between-group abstinence rates at 6 months: Intervention: 27.7%, control: 20.3%, *p =* .315.
Groeneveld et al. (2011) Netherlands Construction	Outcome: Weight, Physical activity, Alcohol, Smoking. I) Over a period of 6 months, 3× 45- to 60-minute face to face and 4× 15- to 30-minute telephone contacts with an occupational physician/occupational nurse. C) Received usual care, consisting of brief oral or written information from the occupational physician about their risk profile.	26 weeks *N =* 816 T1%—82% (*n =* 671); T2%—73% (*n =* 595) 0, 6 m, 12 m	**CO: Between-group smoking cessation rates at 6 months:** Intervention 31.3%, control: 13.4%. Smoking status at 6 months (OR) 0.3 (95% CI: 0.1; 0.7), *p <* .05*. CO: Between-group smoking status differences at 12 months: (OR) 0.8, (95% CI: 0.4; 1.6), *p >* .05.
Alcohol			
Groeneveld et al. (2011) Netherlands Construction	Outcome: Weight, Physical activity, Alcohol, Smoking. I) Over a period of 6 months, 3× 45- to 60-minute face to face and 4× 15- to 30-minute telephone contacts with an occupational physician/occupational nurse. C) Received usual care, consisting of brief oral or written information from the occupational physician about their risk profile.	26 weeks *N =* 816 T1%—82% (*n =* 671); T2%—73% (*n =* 595) 0, 6 m, 12 m	**CO: Between-group alcohol (glasses per week) differences at 6 months:** (β) −1.3 (95% CI: −2.7; 0.1), *p >* .05. CO: Between-group alcohol (glasses per week) differences at 12 months: (β) −1.0 (95% CI: −2.5; 0.4), *p >* .05.
Morgan et al. (2011, 2012) Australia Aluminum manufacture	Outcome: Weight, Physical activity, Diet, Alcohol I) Multicomponent program: (a) information session (75 minutes) providing basic education for weight loss. (b) Study Web site—weight reporting. (c) Pedometer for self-monitoring and as a motivational tool. (d) Two crew-based financial incentives. C) Waitlist.	14 weeks *N =* 110 T1: 82% (*n =* 90) 21 0, 3 m	**ITT: Between-group differences at 14 weeks:** Alcohol risk (AUDIT-C) (x̄) 0.3 (95% CI: −0.7 to 1.2), *p =* .57, effect size (Cohen’s *d*): 0.10.
Viester et al. (2018) Netherlands Construction	Outcome: Weight, Physical activity, Diet, Alcohol I) Nutrition/lifestyle education counseling: face to face and telephone health coaching sessions (2–4) with personalized feedback on health screening; current lifestyle behavior; training instruction, and support in self-monitoring of behavior, goal setting, and evaluation. Focused on improving PA levels and healthy dietary behaviors. C) Usual care.	26 weeks *n =* 314 T2: 83% (*n =* 257) 0, 6 m, 12 m	**CO: Between-group alcohol (glasses per week) differences at 6- months:** (β) = 0.33 (95% CI: ?^a^ to 2.54), *p =* .82. Between-group alcohol (glasses per week) differences at 12- months: (β) = 2.33, (95% CI: 0.90 to 5.56), *p =* .16.
Nutrition			
Choi et al. (2017) South Korea Office-based	Outcome: Weight, Diet I) (a) tai chi + health education—60-min sessions of tai chi exercise twice weekly for 12 weeks (baseline *n =* 34, follow-up *n =* 21) (b) health education only—nurse educator provided the classes separately for each group every 2 weeks. The education topics included (1) definition of metabolic syndrome, (2) modifiable and nonmodifiable risk factors for metabolic syndrome, (3) exercise and an active lifestyle, (4) healthy diet, (5) smoking cessation, and (6) stress management. C) Health education only.	12 weeks *n =* 66 T1: 70% (*n =* 43) 0, 3 m	**CO: Between-group differences at 12 weeks:** Nutrition score *t*=1.98, *p =* .053.
Groeneveld et al. (2011) Netherlands Construction	Outcome: Weight, Physical activity, Alcohol, Smoking. I) Over a period of 6 months, 3× 45- to 60-minute face to face and 4× 15- to 30-minute telephone contacts with an occupational physician/occupational nurse. C) Received usual care, consisting of brief oral or written information from the occupational physician about their risk profile.	26 weeks *N =* 816 T1%—82% (*n =* 671); T2%—73% (*n =* 595) 0, 6 m, 12 m	**CO: Between-group nutrition differences at 6 months:** Vegetables (spoons per week) (β) 0.9 (95% CI: −0.6 to 2.4) *p >* .05. Fruit (pieces per week) (β) 1.7 (95% CI: 0.6 to 2.9), *p <* .05*. Snacks (pieces per week) (β) −1.9 (95% CI: −3.7: −0.02), *p <* .05*. CO: Between-group nutrition differences at 12 months: Vegetables (spoons per week) (β) 0.04 (95% CI: −1.4 to 1.4) *p >* .05. Fruit (pieces per week) (β) 0.9 (95% CI: −0.2 to 2.1) *p >* .05. Snacks (pieces per week) (β) −1.9 (95% CI: −3.6: −0.2) *p <* .05*.
Viester et al. (2018) Netherlands Construction	Outcome: Weight, Physical activity, Diet, Alcohol I) Nutrition/lifestyle education counseling: face to face and telephone health coaching sessions (2–4) with personalized feedback on health screening; current lifestyle behavior; training instruction, and support in self-monitoring of behavior, goal setting, and evaluation. Focused on improving PA levels and healthy dietary behaviors. C) Usual care.	26 weeks *n =* 314 T2: 83% (*n =* 257) 0, 6 m, 12 m	**CO: Between-group differences at 6 months:** SSBs (glasses per week) (β) 2.82 (95% CI: 4.67 to 0.97), *p =* .003*. Snacks (pieces per week) (β) 0.93 (95% CI: 2.66 to 0.80), *p =* .29. Fruit (pieces per week) (β) 1.19 (95% CI: 0.34 to 2.62), *p =* .10. Vegetables (spoons per week) (β) 1.12 (95% CI: 0.48 to 2.72), *p =* .17. CO: Between-group differences at 12 months: SSBs (glasses per week) (β) 0.96 (95% CI: 2.68 to 0.63), *p*=.24. Snacks (pieces per week) (β) 0.63 (95% CI: 2.47 to 1.20), *p =* .50. Fruit (pieces per week) (β) 0.25 (95% CI: 1.44 to 1.94), *p =* .77. Vegetables (spoons per week) (β) 0.62 (95% CI: 1.19 to 2.43), *p =* .50.
Morgan et al. (2011, 2012) Australia Aluminum manufacture	Outcome: Weight, Physical activity, Diet, Alcohol I) Multicomponent program: (1) information session (75 minutes) providing basic education for weight loss. (2) Study Web site—weight reporting. (3) Pedometer for self-monitoring and as a motivational tool. (4) Two crew-based financial incentives. C) Waitlist.	14 weeks *N =* 110 T1: 82% (*n =* 90) 0, 3 m	**ITT: Between-group differences at 14 weeks:** Cola drinks (x̄) 1.2 (95% CI: 0.2 to 2.1), *p =* .02, effect size (Cohen’s *d*): 0.27. Other soda drinks (x̄) 1.4 (95% CI: 0.4 to 2.6), *p =* .01, effect size (Cohen’s *d*): 0.60. Fruit (serves p/day) (x̄) 0.4 (95% CI: −0.0 to 0.8), *p =* .06, effect size (Cohen’s *d*): 0.32. Vegetables (serves p/day) (x̄) 0.2 (95% CI: −0.2 to 0.6), *p =* .39, effect size (Cohen’s *d*): 0.19. Diet drinks (x̄) 0.8 (95% CI: −0.3 to 1.9), *p =* .17, effect size (Cohen’s *d*): 0.27.
Physical activity			
Nicolson et al. (2021) Ireland Office-workers, *n =* 22	Outcome: Physical activity. I) “Cycle at Works” education session on the dangers associated with prolonged sedentary behavior and the potential benefits of reducing sedentary behavior; participants were provided with an under-desk pedal device to enable light physical activity throughout the workday to interrupt sedentary behavior; cycling/pedaling time goals of 30–40 minute per workday C) Waitlist.	2 weeks *n =* 22 T1:95% (*n =* 21) 0, 0.5 m	**CO: Between-group differences at 2 weeks:** Sedentary behavior (min per workday) (x̄) −20.4. Total weekday physical activity (min per day) (x̄) 9.9. Workday standing (min per day) (x̄) 14.4. Total weekday standing (min per day) (x̄) 23. (NB: significance between groups not explored).
Morgan et al. (2011, 2012) Australia Aluminum manufacture	Outcome: Weight, Physical activity, Diet, Alcohol. I) Multicomponent program: (a) information session (75 minutes) providing basic education for weight loss. (b) Study Web site—weight reporting. (c) Pedometer for self-monitoring and as a motivational tool. (d) Two crew-based financial incentives. C) Waitlist.	14 weeks *N =* 110 T1: 82% (*n =* 90) 0, 3 m	**ITT: Between-group differences at 14 weeks:** Total METs (x̄) 0.3, (95% CI: 0.0 to 0.5), *p =* .03*, Effect size (Cohen’s *d*): 0.77. Current PA level (x̄) 0.6 (95% CI: 0.2:1.0), *p <* .001*, effect size (Cohen’s *d*): 0.75.
Kim et al. (2015) South Korea Mixed working environment	Outcome: Weight, Physical activity I) Text message-based application that was tailored to participants ‘individual dietary behaviors and physical activity levels based on responses to questionnaires and metabolic risk profiles that were assessed by laboratory examinations and anthropometric measurements + 4× offline education sessions +brief monthly counseling with nurse. C) Education sessions + brief monthly counseling.	26 weeks *n =* 205 T1: 60% (*n =* 122) 0, 6 m	**ITT: Between-group differences in MET-minutes/week:** 1 month (β) −51.8 (95% CI: −777.3 to 673.7), *p =* .89. 3 months (β) 481.0 (95% CI: −283.3 to 1245.3), *p =* .22. 6 months (β) 580.4 (95% CI: −179.2, 1340.0), *p =* .14.
Gram et al. (2012) Denmark Construction	Outcome: Weight, Physical activity I) Exercise group −3× 20-minute aerobic and strength exercise sessions for 12 weeks C) 1 hour lecture on general health.	12 weeks *n =* 67 T1: 97% (*n =* 63) 0, 3 m	**ITT: Between-group differences after 12 weeks (intervention vs. control):** Vo2max (x̄) 0.4 (95% CI: 0.2 to 0.5), *p <* .001*.
Maruyama et al. (2010) Japan Office-workers	Outcome: Weight, Physical activity I) Monthly individual contact with a dietitian and a physical trainer, collaborative goal setting sessions based on food group intake and physical activity and received monthly website advice C) No intervention.	16 weeks *n =* 101 T1: 86% (*n =* 87) 0, 4 m	**CO: Between-group differences at 16 weeks:** Walking steps (x̄) 942 (95% CI: −379 to 2262), *p =* .16, effect size (Cohen’s *d*): 0.91.
Viester et al. (2018) Netherlands Construction	Outcome: Weight, Physical activity, Diet, Alcohol I) Nutrition/lifestyle education counseling: face to face and telephone health coaching sessions (2–4) with personalized feedback on health screening; current lifestyle behavior; training instruction, and support in self-monitoring of behavior, goal setting, and evaluation. Focused on improving PA levels and healthy dietary behaviors. C) Usual care.	26 weeks *n =* 314 T2: 83% (*n =* 257) 0, 6 m, 12 m	**CO: Between-group difference at 6 months:** Leisure time MVPA (min per week) (β) 70.6 (95% CI: 24.3 to 165.5), *p =* .14. Public health guideline VPA (%) (β) 2.06 (95% CI: 1.07 to 3.99), *p =* .032*. CO: Between-group difference at 12 months: Leisure time MVPA (min per week) (β) 27.0 (95% CI: 104.7 to 50.7), *p =* .49. Public health guideline VPA (%) (β) 1.52 (95% CI: 0.81 to 2.83), *p =* .19.
Groeneveld et al. (2011) Netherlands Construction *N* = 816	Outcome: Weight, Physical activity, Alcohol, Smoking I) Over a period of 6 months, 3× 45- to 60-minute face to face and 4× 15- to 30-minute telephone contacts with an occupational physician/occupational nurse. C) Received usual care, consisting of brief oral or written information from the occupational physician about their risk profile.	26 weeks *N =* 816 T1%—82% (*n =* 671); T2%—73% (*n =* 595) 0, 6 m, 12 m	**CO: Between-group differences at 6 months:** Leisure time physical activity (minutes per week) (β) 59.5 (95% CI: −11.3 to 130.3), *p >* .05. Sports activities (minutes per week) (β) 10.1 (95% CI: −9.6 to 29.7), *p >* .05. MET (minutes per week) (β) 226.5 (95% CI: −81.6 to 534.5), *p >* .05. CO: Between-group differences at 12 months: Leisure time physical activity (minutes per week) (β) 30.2 (−45.3 to 105.8), *p >* .05. Sports activities (minutes per week) (β) 2.2 (95% CI: −19.0 to 23.5), *p >* .05. MET (minutes per week) (β) 132.2 (95% CI: −177.7 to 442.0), *p >* .05.
Weight outcomes			
Iriyama & Murayama (2014) Japan Office-based	Outcome: Weight, Diet I) Nutrition education session including health education sessions, individual counseling and self-help guide booklet & food environment intervention including healthy menus (defined as a meal containing 600–700 kcal of energy and ≥120 g of vegetables, with a fat/energy ratio of 20%–25%) were served only to the intervention group. C) Waitlist.	26 weeks *n =* 79 T1: 82% (*n =* 65), T2: 72% (*n =* 57) 0,6 m	**CO: Between-group differences at 1 year:** Weight (kg): Intervention (x̄) −1.6, control (x̄) 0.0, *p =* .017*. BMI (kg/m²): Intervention (x̄) −0.8, control (x̄) −0.2, *p =* .017*. WC (cm): Intervention (x̄) −1.3, control (x̄) −0.9, *p >* .05.
Choi et al. (2017) South Korea Office-based	Outcome: Weight, Diet I) (a) tai chi + health education—60-min sessions of tai chi exercise twice weekly for 12 weeks (baseline *n =* 34, follow-up *n =* 21) (b) health education only—nurse educator provided the classes separately for each group every 2 weeks. The education topics included (1) definition of metabolic syndrome, (2) modifiable and nonmodifiable risk factors for metabolic syndrome, (3) exercise and an active lifestyle, (4) healthy diet, (5) smoking cessation, and (6) stress management. C) Health education only.	12 weeks *n =* 66 T1: 70% (*n =* 43) 0, 3 m	**CO: Between-group differences at 12 weeks:** WC (cm), *t* = −1.38 *p =* .17.
Gram et al. (2012) Denmark Construction	Outcome: Weight, Physical activity I) Exercise group − 3× 20-minute aerobic and strength exercise sessions for 12 weeks C) 1-hour lecture on general health.	12 weeks *n =* 67 T1: 97% (*n =* 63) 0,3-m	**ITT: Between-group differences after 12 weeks:** Weight (kg) (x̄) 0.3, (95% CI: −1.0 to 1.5), *p =* .68. BMI (kg/m²): (x̄) 0.1 (95% CI: −0.3 to 0.6), *p =* .55. Percent body fat (%): (x̄) 2.9 (95% CI: −3.6 to 9.4), *p =* .37.
Groeneveld et al. (2011) Netherlands Construction	Outcome: Weight, Physical activity, Alcohol, Smoking I) Over a period of 6 months, 3× 45- to 60-minute face to face and 4× 15- to 30-minute telephone contacts with an occupational physician/occupational nurse. C) Received usual care, consisting of brief oral or written information from the occupational physician about their risk profile.	26 weeks *N =* 816 T1%—82% (*n =* 671); T2%—73% (*n =* 595) 0, 6 m, 12 m	**CO: Between-group differences at 6 months:** Bodyweight (kg) (β) −1.9 (95% CI: −2.6: −1.2), *p <* .05*. BMI (kg/m^2^) (β) −0.6 (95% CI: −0.8: −0.3), *p <* .05*. CO: Between-group differences at 12 months: Bodyweight (kg) (β) −1.8 (95% CI: −2.6: −1.1), *p <* .05*. BMI (kg/m^2^) (β) −0.6 (95% CI: −0.8: −0.3), *p <* .05*.
Maruyama et al. (2010) Japan Office-workers	Outcome: Weight, Physical activity I) Monthly individual contact with a dietitian and a physical trainer, collaborative goal setting sessions based on food group intake and physical activity and received monthly website advice C) No intervention.	16 weeks *n =* 101 T1: 86% (*n =* 87) 0,4 m	**CO: Between-group differences at 16 weeks:** Weight (kg) (x̄) −1.29 (95% CI: −2.32: −0.27), *p =* .01*, effect size (Cohen’s *d*): 0.13. BMI (kg/m²) (x̄) −0.47 (95% CI: −0.82: −0.11), *p =* .01*, effect size (Cohen’s *d*): 0.15.
Morgan et al. (2011, 2012) Australia Aluminum manufacture	Outcome: Weight, Physical activity, Diet, Alcohol. I) Multicomponent program: (a) information session (75 minutes) providing basic education for weight loss. (b) Study Web site—weight reporting. (c) Pedometer for self-monitoring and as a motivational tool. (d) Two crew-based financial incentives. C) Waitlist.	14 weeks *N =* 110 T1: 82% (*n =* 90) 0, 3-m	**ITT: Between-group differences at 14 weeks:** Body weight (kg) (x̄) 4.3 (95% CI: 2.6 to 6.1), *p <* .001*, effect size (Cohen’s *d*): 0.34. BMI (kg/m²) (x̄) 1.4 (95% CI: 0.9 to 2.0), *p <* .001*, effect size (Cohen’s *d*): 0.41. WC (cm) (x̄) 5.9 (95% CI: 4.2 to 7.6), *p <* .001*, effect size (Cohen’s *d*): 0.63.
Lim et al. (2015) South Korea Mixed	Outcome: Weight. I) Exercise group—10-week exercise program intervention consisted of 3 days of walking exercise per week for 30 minute/day. C) No intervention.	10 weeks *n =* 30 T1=100% (*n =* 30) 0, 2.5 m	**CO: Within group differences at 10 weeks:** Weight (kg): Intervention (x̄) −0.37, control (x̄) 0.11, between-group significance *p =* .005*. BMI (kg/m^2^): Intervention (x̄) −0.10, control (x̄) 0.04, between-group significance *p =* .091. Fat mass (kg): Intervention (x̄) −0.13, control (x̄) 0.09, between-group significance *p =* .002*. Percent body fat (%): Intervention (x̄) −0.07, control (x̄) 0.10, between-group significance: *p =* .004*. Fat free mass (kg): Intervention (x̄) −0.24, control (x̄) 0.02, between-group significance *p =* .29.
Kim et al. (2015) South Korea Mixed working environments	Outcome: Weight, Physical activity I) Text message-based application that was tailored to participants ‘individual dietary behaviors and physical activity levels based on responses to questionnaires and metabolic risk profiles that were assessed by laboratory examinations and anthropometric measurements + 4× offline education sessions +brief monthly counseling with nurse. C) Education sessions + brief monthly counseling.	26 weeks *n =* 205 T1: 60% (*n =* 122) 0, 6 m	**ITT: Between-group differences at 1 month** Weight (kg) (β) −1.07 (95% CI: −1.85: −0.30), *p =* .01*. Percent body fat (β) −1.09(95% CI: −2.60 to 0.43), *p =* .16. ITT: Between-group differences at 3 months: Weight (kg) (β) −0.18 (95% CI: −1.15 to 0.79), *p =* .85. Percent body fat (β) −0.19 (95% CI: −1.64 to 1.25), *p =* .80. ITT: Between-group differences at 6 months: Weight (kg) (β) −0.15 (95% CI: −1.36 to 1.07), *p =* .78. Percent body fat (β) 0.28 (95% CI: −0.75 to 1.31), *p =* .60.
Viester et al. (2018) Netherlands Construction	Outcome: Weight, Physical activity, Diet, Alcohol I) Nutrition/lifestyle education counseling: face to face and telephone health coaching sessions (2–4) with personalized feedback on health screening; current lifestyle behavior; training instruction, and support in self-monitoring of behavior, goal setting, and evaluation. Focused on improving PA levels and healthy dietary behaviors. C) Usual care.	26 weeks *n =* 314 T2: 83% (*n =* 257) 0, 6-m, 12-m	**CO: Between-group differences at 6 months:** Weight (kg) (β) −1.06 (95% CI: 1.87 to 0.26), *p =* .01*. BMI (kg/m²) (β) −0.32 (95% CI: −0.57, −0.08) *p =* .01*. WC (cm) (β) −1.38 (95% CI: −2.63 to 0.12), *p =* .032*. **CO: Between-group differences at 12 months:** Weight (kg) (β) −1.00 (95% CI: −2.01 to 0.01), *p =* .053. BMI (kg/m²) (β) −0.30 (95% CI: −0.61 to 0.01), *p =* .06. WC (cm): (β) −0.91 (95% CI: −2.25 to 0.44), *p =* .19.

*Note.* ITT = intention to treat; CO = completers only; OR = odds ratio; CI = confidence interval; AUDIT-C = Alcohol Use Disorders Identification Test for Consumption; BMI = body mass index; BF% = body fat percentage; FFQ = Food Frequency Questionnaire; RCT = randomized controlled trial; T0 = time point zero; T1 = time point 1; T2 = time point 2; T3 = time point 3; PA = physical activity; SSB = sugar-sweetened beverage; MET = metabolic equivalent of task; MVPA = moderate-to-vigorous physical activity; NRT = nicotine replacement therapy; WC = waist circumference; NB = Nota bene. Study characteristics include intervention duration, *N* at baseline, retention percentages at timepoints, and data collection timepoints.

a Viester: This value was missing from the manuscript published.

* Statistically significant.

Of all included studies, eight reported a weight-related outcome (Choi et al., 2017; Gram et al., 2012; Iriyama & Murayama, 2014; Kim et al., 2015; Lim et al., 2015; Maruyama et al., 2010; Morgan et al., 2011; Viester et al., 2018). This included six studies from the bodyweight meta-analysis and five from the BMI meta-analysis. Of the eight studies, five studies report a significant effect favoring the intervention for bodyweight (Groeneveld et al., 2011; Lim et al., 2015; Maruyama et al., 2010; Morgan et al., 2011; Viester et al., 2018). Three studies reported a significant effect regarding BMI (Maruyama et al., 2010; Morgan et al., 2011; Viester et al., 2018), and two studies reported a significant effect for waist circumference (Morgan et al., 2011; Viester et al., 2018). Iriyama and Murayama (2014) reported statistically significant results for both bodyweight and BMI; however, these results were produced both in the control and intervention group. The remaining three studies reported no statistical differences (Choi et al., 2017; Gram et al., 2012; Kim et al., 2015).

Four studies reported nutrition-related outcomes (Choi et al., 2017; Groeneveld et al., 2011; Morgan et al., 2011; Viester et al., 2018). One study demonstrated a significant between**-**group difference regarding snacking frequency favoring the intervention (Groeneveld et al., 2011). Two studies showed a significant between-group difference favoring the intervention regarding sugar-sweetened beverage consumption (Morgan et al., 2011; Viester et al., 2018). One study was able to illustrate a significant intervention effect when compared with control regarding increased fruit consumption at 6 months; however, this effect was not sustained at the 12-month follow-up (Groeneveld et al., 2011). Across the four studies, there were no statistically significant improvements to vegetable intake between groups.

Two studies reported smoking cessation outcomes (Asfar et al., 2020; Groeneveld et al., 2011). Groeneveld et al. (2011) did show a significant between-group difference regarding smoking cessation at 6 months; however, this was not sustained at the 12-month follow-up. Asfar et al. (2020) reported no significant between-group differences regarding prolonged abstinence.

Three studies reported alcohol-related outcomes (Groeneveld et al., 2011; Morgan et al., 2011; Viester et al., 2018) and the direction of the relationship was encouraging. Groeneveld et al. (2011) and Viester et al. (2018) illustrated a mean decrease in the number of alcoholic drinks per week (glass per week). Morgan et al. (2011) reported overall improvements to risky/hazardous drinking behaviors, although across the three studies, results were not significant.

### Weight: Meta-Analysis

**Figure 2:**
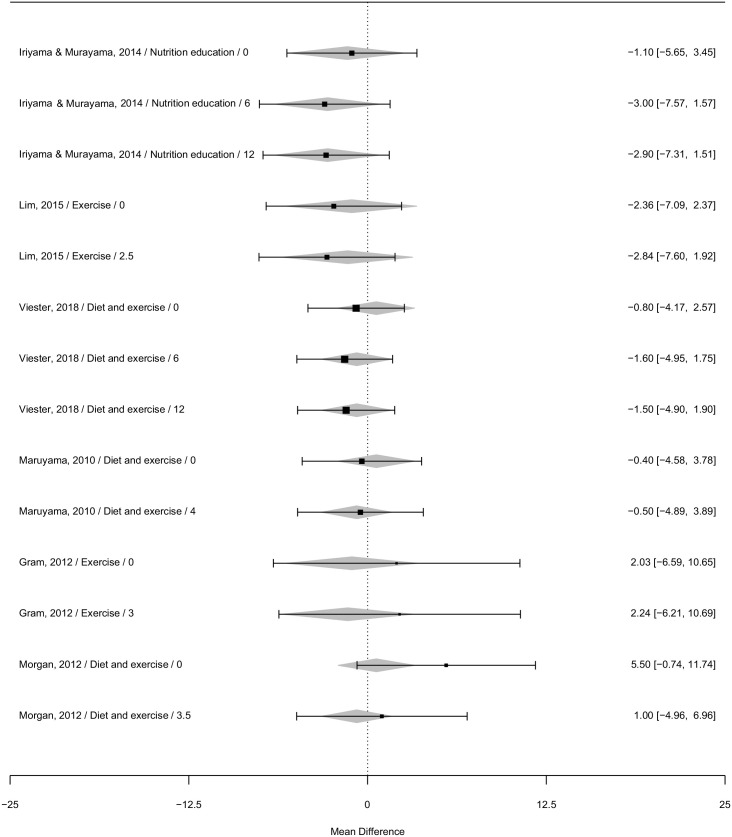
Forest Plot of Mean Differences of Included Studies Reporting Body Weight (kg) Outcomes and 95% CI at All Timepoints (Author/Year/Intervention/Time Point (Months) *Note.* CI = confidence interval; LRT = likelihood ratio test (goodness-of-fit).

Meta-analysis of body weight (kg) included six studies, with a total of six intervention arms targeting weight and examined two moderator effects. The forest plots (Figure 2) highlight the varying effect sizes, mean differences, and respective confidence intervals for each included study over time. Overall, there was a nonsignificant time effect, LRT χ^2^(4) = 1.12, *p* = .57, with a mean decrease in weight relative to baseline of −0.28 kg bodyweight up to 3 months (95% CI: −6.15 to 5.58) and −1.38 kg bodyweight for >3 months (95% CI: −4.11 to 1.36) (Supplementary Material 7) in the intervention group compared with control. The funnel plot (Supplementary Material 8) demonstrated symmetry, indicating there was no evidence of publication bias to higher values, a nonparametric correlation test supported this (Kendall’s tau 0.28, *p* = .19). Model diagnostics were satisfactory and are in Supplementary Material 9.

### BMI: Meta-Analysis

**Figure 3.**
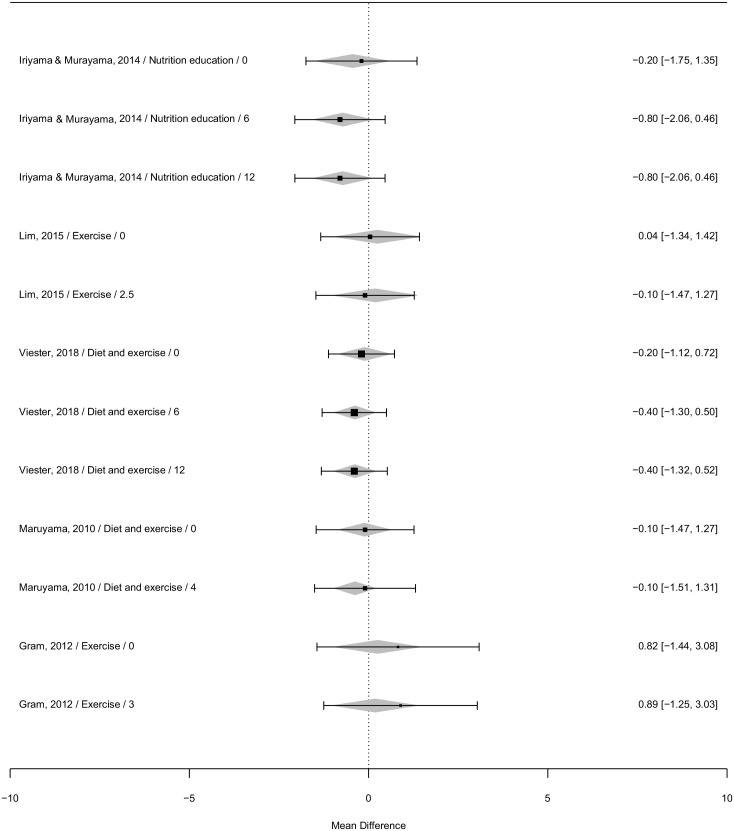
Forest Plot of Mean Differences of Included Studies Reporting BMI (kg/m^2^) Outcomes and 95% CI at All Timepoints (Author/Year/Intervention/Time Point (Months) *Note.* BMI = body mass index; CI = confidence interval.

Meta-analysis of BMI (kg/m^2^) included five studies, with a total of five intervention arms targeting BMI and examined two moderator effects. The forest plots regarding BMI highlight the varying effect sizes, mean differences, and respective confidence intervals for each included study over time (Figure 3). Overall, there was a nonsignificant time effect, LRT χ^2^(4) = 0.41, *p* = .82, with a mean decrease in BMI relative to baseline of −0.06 kg/m^2^ up to 3 months (95% CI: −1.71 to 1.59) and −0.27 kg/m^2^ for >3 months (95% CI: −1.12 to 0.57) (Supplementary Material 10) in the intervention group compared with control. The funnel plot (Supplementary Material 11) demonstrated non-symmetrical distribution, indicating there is evidence of publication bias to higher values, a nonparametric correlation test supported this (Kendall’s tau 0.50, *p* = .02). Model diagnostics were satisfactory and are in Supplementary Material 12.

## Discussion

The aim of this systematic review and meta-analysis was to evaluate the effectiveness of WHP programs targeting smoking, nutrition, alcohol, physical activity, and/or overweight and obesity risk factors in men. To the authors’ knowledge, this is the first review to investigate this topic area exclusively in males. Workplace health programs have had increased attention since 2010 (Peñalvo et al., 2021), prompting a more contemporary examination into the literature. The results of this review highlight those interventions targeting overweight and obesity, physical activity, sugar-sweetened beverage and snack consumption, have demonstrated encouraging results. Contrastingly, aspects around fruit and vegetable consumption, smoking cessation, and alcohol consumption did not produce any statistically significant between-group differences. These results underscore the difficulty of modifying health behaviors within the context of the workplace in males.

Changing men’s health behaviors and attitudes around fruit, vegetable and alcohol consumption is challenging. This phenomenon could be associated with masculine ideologies and values (Verdonk et al., 2010). Hegemonic masculinity norms reinforce the notion that men tend to distance themselves from the feminized realm of dieting (Gough, 2007). This may account for why weight interventions that incorporate physical activity components tend to produce greater treatment effects and have better retention in men (Bezzina et al., 2022). Moreover, patterns of conformity and peer pressure around alcohol consumption are particularly relevant for higher alcohol consumption within male dominated industries (Roberts et al., 2019). This is further reinforced by this review, whereby the three studies that investigated alcohol consumption were all in industries and settings that are traditionally male dominated (construction and manufacturing).

One theme that emerged during data analysis was the interplay between weight loss programs and the use of individualized dietary feedback. Interestingly, the three low-risk-of-bias studies that employed this individualized approach all produced statistically significant weight loss compared with the control group (Iriyama & Murayama, 2014; Maruyama et al., 2010; Morgan et al., 2012). Through a behavior change lens, individualized dietary feedback incorporates aspects of self-monitoring and recording a behavior. Self-monitoring is a powerful mechanism for change and has been associated with successful weight loss interventions (Dombrowski et al., 2012). A 2012 systematic review and meta-analysis that examined behavioral interventions for adults with obesity reinforces this notion (Dombrowski et al., 2012). With regard to weight effects for specific dietary behavior-change techniques, prompting self-monitoring of behavior was significantly associated with greater mean difference in weight loss between control and intervention groups (β = −3.37, *p* = .001) (Dombrowski et al., 2012). Technology integrated automated personalized nutrition feedback systems could further streamline this process and have shown promising signs in clinical settings (Zenun Franco et al., 2022). This could reduce the resource burden of this approach, and further increase its application through workplace health programs.

Meta analytic results in this review highlighted that the mean decrease in weight relative to baseline was −0.28 kg up to 3 months, and −1.38 kg for >3 months across the six included weight studies (*p* = .57). Interestingly, among mixed gender systematic review and meta-analysis’, WHP programs have shown statistically beneficial pooled effects for body weight loss. Peñalvo et al. (2021) recently conducted a systematic review and meta-analysis assessing the effectiveness of workplace wellness programs for dietary habits, overweight, and cardiometabolic health. The review included 121 studies and found that workplace wellness programs significantly reduced BMI (−0.22 kg/m^2^ [95% CI −0.28 to −0.17]), bodyweight (−0.92 kg [−1.11 to −0.72]), and waist circumference (−1.47 cm [−1.96 to −0.98]). Possible explanations regarding the statistical differences among pooled results between this review and Peñalvo et al. (2021) include studies that were mixed gender, including quasi experimental design papers as opposed to randomized controlled trial (RCTs) only, and a notably longer mean duration of interventions (10 months compared with 4 months for this review). Despite the statistical difference, the clinical significance of modest weight loss should not be ignored. This is especially relevant considering global obesity rates have nearly tripled since 1975 (WHO, 2021), and how annual global weight gain for adults ranges from 0.3 to 0.8 kg/year (Edward et al., 2015). Looking closer to males specifically, in OECD countries, men are more likely to be overweight or obese. Reports suggest that gender differences in overweight and obesity status can range up to 14% to 16% in countries like Australia, Germany, and Hungary (OECD, 2021). Therefore, strategies that look to curb chronic habitual weight gain in men are an important first step in fighting the rising prevalence of overweight and obesity and narrowing health inequalities between males and females.

One aspect which ought to be considered when evaluating WHP programs is the role of organizational culture in an individual’s wellness journey. There are numerous definitions of organizational culture, although broadly it can be considered the shared values, beliefs, or perceptions of employees within an organization (Schein, 2010). When it comes to cultivating a culture of health, there are several factors to consider, including leadership support, peer support, and employee engagement. Interestingly, in a study of 825 matched employees across a 18-month period, Payne et al. (2018) found that only leadership support predicted both perceived employee organizational support for health and improved self-reported lifestyle risk factors (*p <* .001) including nutrition, physical activity, and tobacco use (Payne et al., 2018). Leaders who foster a culture of wellness can have a significant impact on the sustainability and effectiveness of WHP programs. This is through means of allocating resources to support programs, providing opportunities for employees to adopt healthy lifestyles (such as allowing time and flexibility to use these programs), and being positive health ambassadors through both actions and words (Kent et al., 2016).

Culture is not only limited to an organization, as the social practices and behavior norms of individual countries can also implicate a person’s wellness. To account for this and to increase the generalizability of results, this review only examined research carried out in OECD countries. However, it would be disingenuous to conclude that all OECD countries have similar workplace practices, especially when considering the varied number of hours worked yearly between regions. This notion is exemplified when comparing the two most represented countries in this review, South Korea (*n* = 3), and the Netherlands (*n* = 2). On average, individuals in South Korea work 26% more hours over a year compared with their Dutch counterparts or 1,915 hours per year compared with 1,417, respectively (OECD, 2023). Anecdotal, but this increased time within the workplace may underpin why interventions conducted in South Korea (Choi et al., 2017; Kim et al., 2015; Lim et al., 2015) leaned more heavily into intervention aspects that were face to face, because of the increased contact hours with employees. In juxtaposition, telephone coaching sessions and prompting of self-monitoring were synonymous with studies conducted in the Netherlands (Groeneveld et al., 2011; Viester et al., 2018), underscoring the need for caution when generalizing results, and planning future wellness interventions.

The strength of this review includes a comprehensive search strategy targeting several modifiable health behaviors, two independent reviewers at each of the review stages, utilization of the Cochrane Collaboration’s Tool for assessing risk of bias, and a robust statistical analysis process. While this review wanted to examine contemporary literature, this could be perceived as a limitation. As there were fewer studies included that examined effects concerning smoking, nutrition, alcohol, and physical activity, in conjunction with different reporting methods (serves per day compared with pieces per week, etc.), a meta-analysis concerning these variables was not feasible. This review is the first to focus on WHP programs in men only; however, this means that results may not be transferrable to all workplaces or those primarily made up of females.

## Conclusions

This systematic review and meta-analysis highlight the potential of WHP programs targeting certain modifiable health behaviors in males, however evidence concerned with fruit, vegetable, and alcohol consumption, as well as smoking cessation was less promising. While weight outcomes did not produce statistically significant results, the direction of the relationship is encouraging, especially in the context of work, health, and safety more broadly. More high-quality studies are needed to determine long-term effectiveness of WHP programs targeting smoking, nutrition, alcohol, physical activity, and/or overweight and obesity in men. Understanding the workplace environment through a social practice lens, rather than solely focusing on the individual behavior journey, may prove more fruitful in changing men’s attitudes and beliefs toward alcohol awareness, fruit, and vegetable consumption. Understanding the effectiveness of novel programs targeting modifiable health behaviors in men is just one step in reducing the burden of chronic disease.

## Supplemental Material

sj-docx-1-heb-10.1177_10901981231208396 – Supplemental material for Workplace Health Promotion Programs Targeting Smoking, Nutrition, Physical Activity, and Obesity in Men: A Systematic Review and Meta-Analysis of Randomized Controlled Trials Click here for additional data file. Supplemental material, sj-docx-1-heb-10.1177_10901981231208396 for Workplace Health Promotion Programs Targeting Smoking, Nutrition, Physical Activity, and Obesity in Men: A Systematic Review and Meta-Analysis of Randomized Controlled Trials by Aaron Bezzina, Erin D. Clarke, Lee Ashton, Trent Watson and Carole L. James in Health Education & Behavior
