# Supplementary material for: Workplace Health Promotion Programs Targeting Smoking, Nutrition, Physical Activity, and Obesity in Men: A Systematic Review and Meta-Analysis of Randomized Controlled Trials
Source: Health Educ Behav. 2023 Nov 27;51(1):113–27. doi: 10.1177/10901981231208396 (PMC10785566; doi:10.1177/10901981231208396)
Supplement: sj-docx-1-heb-10.1177_10901981231208396 – Supplemental material for Workplace Health Promotion Programs Targeting Smoking, Nutrition, Physical Activity, and Obesity in Men: A Systematic Review and Meta-Analysis of Randomized Controlled Trials [file sj-docx-1-heb-10.1177_10901981231208396.docx]

Supplementary Materials 1: Medline Search strategy

Database(s): Ovid MEDLINE(R) and Epub Ahead of Print, In-Process, In-Data-Review & Other Non-Indexed Citations and Daily 1946 to August 25, 2021

| **#** | **Searches** | **Results** |
| --- | --- | --- |
| 1 | Workplace/ or Work/ or Occupational Health/ or Occupational Medicine/ | 94987 |
| 2 | (Workplace or worksite or office).tw. | 107536 |
| 3 | Randomized Controlled Trial/ or Controlled Clinical Trial/ or Clinical Trials as Topic/ or Random Allocation/ or Evaluation Studies/ or Comparative Study/ or Systematic Review/ | 2925872 |
| 4 | review.tw. | 1802369 |
| 5 | random*.tw. | 1247120 |
| 6 | trial.tw. | 657973 |
| 7 | groups.tw. | 2264120 |
| 8 | experiment*.tw. | 2182231 |
| 9 | (pretest or pre test or posttest or post test).tw. | 31872 |
| 10 | intervention*.tw. | 1109747 |
| 11 | program*.tw. | 943891 |
| 12 | control*.tw. | 4120887 |
| 13 | Obesity/ | 192895 |
| 14 | Weight Gain/ | 32866 |
| 15 | Weight Loss/ | 39107 |
| 16 | obes*.tw. | 326084 |
| 17 | (weight gain or weight loss).tw. | 154540 |
| 18 | (overweight or over weight or overeat* or over eat*).tw. | 78764 |
| 19 | weight change*.tw. | 12114 |
| 20 | ((bmi or body mass index) adj2 (gain or loss or change)).tw. | 5136 |
| 21 | (obesity adj2 (prevent* or treat*)).tw. | 21795 |
| 22 | Exercise/ | 122313 |
| 23 | physical inactivity.tw. | 8730 |
| 24 | physical activity.tw. | 120287 |
| 25 | Physical Fitness/ | 28360 |
| 26 | sedentary.tw. | 33985 |
| 27 | (exercise* adj aerobic*).tw. | 247 |
| 28 | Diet/ | 169879 |
| 29 | nutrition*.tw. | 297481 |
| 30 | healthy eating.tw. | 8133 |
| 31 | fruit*.tw. | 118981 |
| 32 | vegetable*.tw. | 60978 |
| 33 | dietary intake.tw. | 26320 |
| 34 | soft drink*.tw. | 4043 |
| 35 | soda.tw. | 4433 |
| 36 | sweetened drink*.tw. | 326 |
| 37 | Alcohols/ | 15722 |
| 38 | Alcohol Drinking/ | 70285 |
| 39 | Alcohol Abuse/ | 76915 |
| 40 | alcohol*.tw. | 353298 |
| 41 | binge.tw. | 14458 |
| 42 | Smoking Prevention/ or Smoking Cessation/ or Smoking Reduction/ | 42340 |
| 43 | ((smok* or nicotine or tobacco) adj2 (cessation or prevent* or stop or quit* or abstain or reduc*)).tw. | 46712 |
| 44 | (Men or male).tw. | 1397951 |
| 45 | 1 or 2 | 185466 |
| 46 | 3 or 5 or 6 or 7 or 8 or 9 or 10 or 11 or 12 | 10432926 |
| 47 | 13 or 14 or 15 or 16 or 17 or 18 or 19 or 20 or 21 or 22 or 23 or 24 or 25 or 26 or 27 or 28 or 29 or 30 or 31 or 32 or 33 or 34 or 35 or 36 or 37 or 38 or 39 or 40 or 41 or 42 or 43 | 1596064 |
| 48 | 44 and 45 and 46 and 47 | 1621 |
| 49 | limit 48 to (english language and yr="2010 -Current") | 753 |

Supplementary Materials 2: Summary of study characteristics from 11 interventions targeting chronic disease risk factors.

| Outcome | Sub-categories | Total^a^ (n=11) | Smoking^b^ (n=2) | Nutrition ^b^ (n=4) | Alcohol ^b^  (n=3) | PA ^b^  (n=7) | OW/OB ^b^  (n=8) |
| --- | --- | --- | --- | --- | --- | --- | --- |
| Publication year | 2010 - 2014 | 5 | 1 | 2 | 2 | 4 | 5 |
|  | 2015 - 2019 | 3 | 0 | 2 | 1 | 2 | 4 |
|  | 2020 - 2021 | 3 | 1 | 0 | 0 | 1 | 0 |
| Country | United states | 1 | 1 | 0 | 0 | 0 | 0 |
|  | Australia | 1 | 0 | 1 | 1 | 1 | 1 |
|  | Netherlands | 2 | 1 | 2 | 2 | 2 | 2 |
|  | South Korea | 3 | 0 | 1 | 0 | 1 | 3 |
|  | Japan | 2 | 0 | 0 | 0 | 1 | 2 |
|  | Ireland | 1 | 0 | 0 | 0 | 1 | 0 |
|  | Denmark | 1 | 0 | 0 | 0 | 1 | 1 |
| Number of participants | Total | 1,932 | 950 | 1,306 | 1,240 | 1,635 | 1,788 |
|  | Mean | 176 | 475 | 327 | 413 | 234 | 199 |
|  | Median | 101 | 475 | 212 | 314 | 110 | 101 |
|  | Range | 22 –  816 | 134-  816 | 66 –  816 | 110 - 816 | 22- 816 | 30 –  816 |
| Setting | Construction | 4 | 2 | 2 | 2 | 3 | 2 |
|  | Manufacturing | 1 | 0 | 1 | 1 | 1 | 1 |
|  | Office-based | 4 | 0 | 1 | 0 | 2 | 3 |
|  | Mixed | 2 | 0 | 0 | 0 | 1 | 1 |
| Intervention duration | Mean duration, months | 4 | 4 | 4.75 | 5.25 | 4.25 | 4.5 |
|  | Range | 0.5 - 6 | 2 – 6 | 3 - 6 | 3.5 - 6 | 0.5 - 6 | 2.5 - 6 |
|  | 0 to ≤3-months, n, % | 5 | 1 | 1 | 0 | 2 | 3 |
|  | 3.25 to ≤6-months, n, % | 6 | 1 | 3 | 3 | 4 | 4 |
| Length of follow-up from end of intervention | Number of studies with follow-ups | 3 | 2 | 2 | 2 | 2 | 2 |
|  | Mean length, months | 5 | 4.5 | 6 | 6 | 6 | 6 |
|  | Range | 3 - 6 | 3 - 6 | 6 | 6 | 6 | 6 |
|  | 0 to ≤3-months, n, % | 1 | 1 | 0 | 0 | 0 | 0 |
|  | 4 to ≤6-months, n, % | 2 | 1 | 2 | 2 | 2 | 0 |
| Retention rate % | Post-intervention, mean % | 92 | 77 | 84 | 79.3 | 82 | 71 |
|  | Range % | 60 - 100 | 70 - 83 | 73 - 97 | 73 - 83 | 60 - 97 | 60 - 100 |
| Theory | Social cognitive theory | 4 | 1 | 1 | 1 | 1 | 1 |
|  | Transtheoretical model | 2 | 1 | 1 | 1 | 1 | 1 |
|  | Socio-ecological theory | 1 | 0 | 0 | 0 | 1 | 0 |
|  | Intervention mapping | 1 | 0 | 1 | 1 | 1 | 1 |
| Primary Intervention focus | Smoking cessation | 1 |  |  |  |  |  |
|  | Nutrition | 0 |  |  |  |  |  |
|  | Alcohol | 0 |  |  |  |  |  |
|  | Physical activity | 3 |  |  |  |  |  |
|  | Weight control / weight loss | 4 |  |  |  |  |  |
|  | Broad wellness | 3 |  |  |  |  |  |
| **Notes:**  ^a^Values outlined in the ‘Total’ column are a frequency of all included studies (n=11).  ^b^Values outlined in the individual chronic disease risk factors are representative of studies that reported on that specific outcome. Noting that multiple studies reported on multiple outcomes, hence the cumulative difference between the total column, and the sum of the individual columns.  Physical Activity (PA), Overweight / Obesity (OW/OB) | | | | | | | |

Supplementary Materials 3: Percentage of intention to treat studies from risk of bias assessment that were categorised as low, high, or unclear risk for individual risk components.

Supplementary Materials 4: Risk of bias individual study assessments across the five different categories – Intension to Treat Studies.

Supplementary Materials 5: Percentage of per protocol studies from risk of bias assessment that were categorised as low, high, or unclear risk for individual risk components.

Supplementary Materials 6: Risk of bias individual study assessments across the five different categories – Per Protocol Studies.


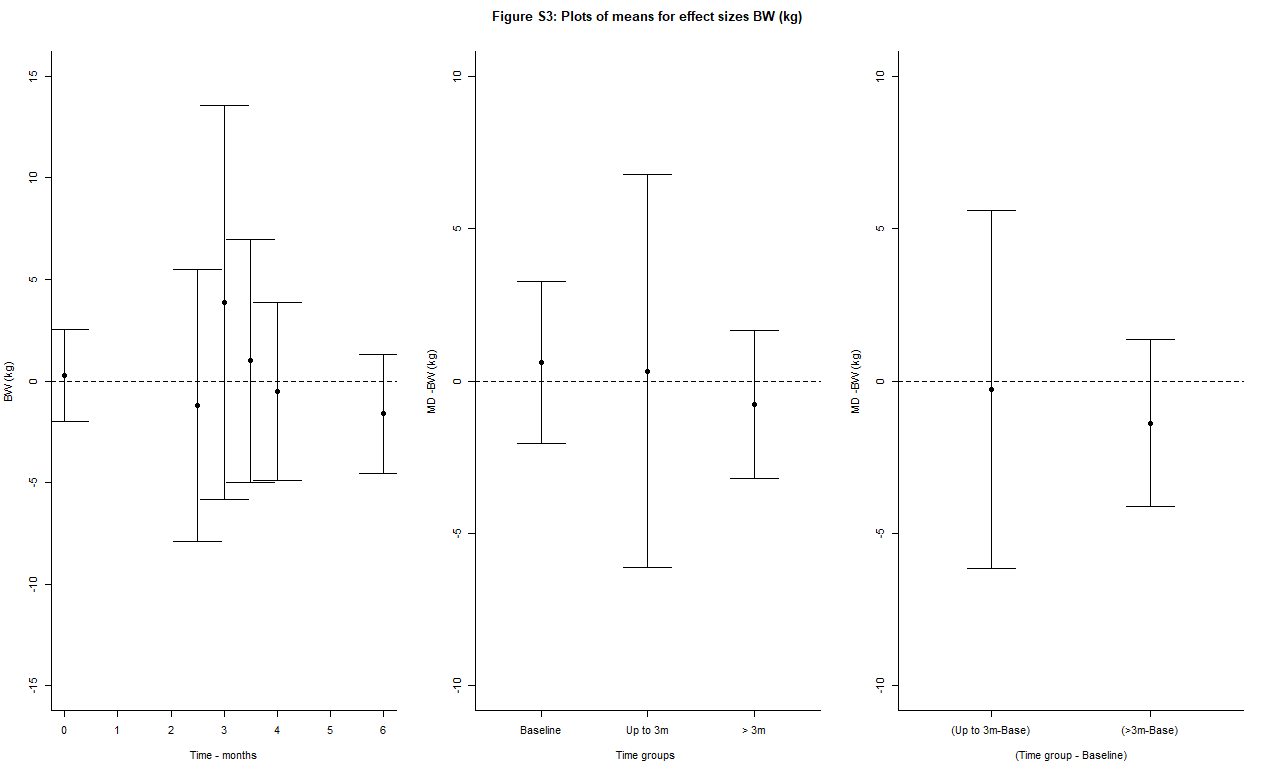


Supplementary Materials 7: Plots of means for effect sizes bodyweight (kg).


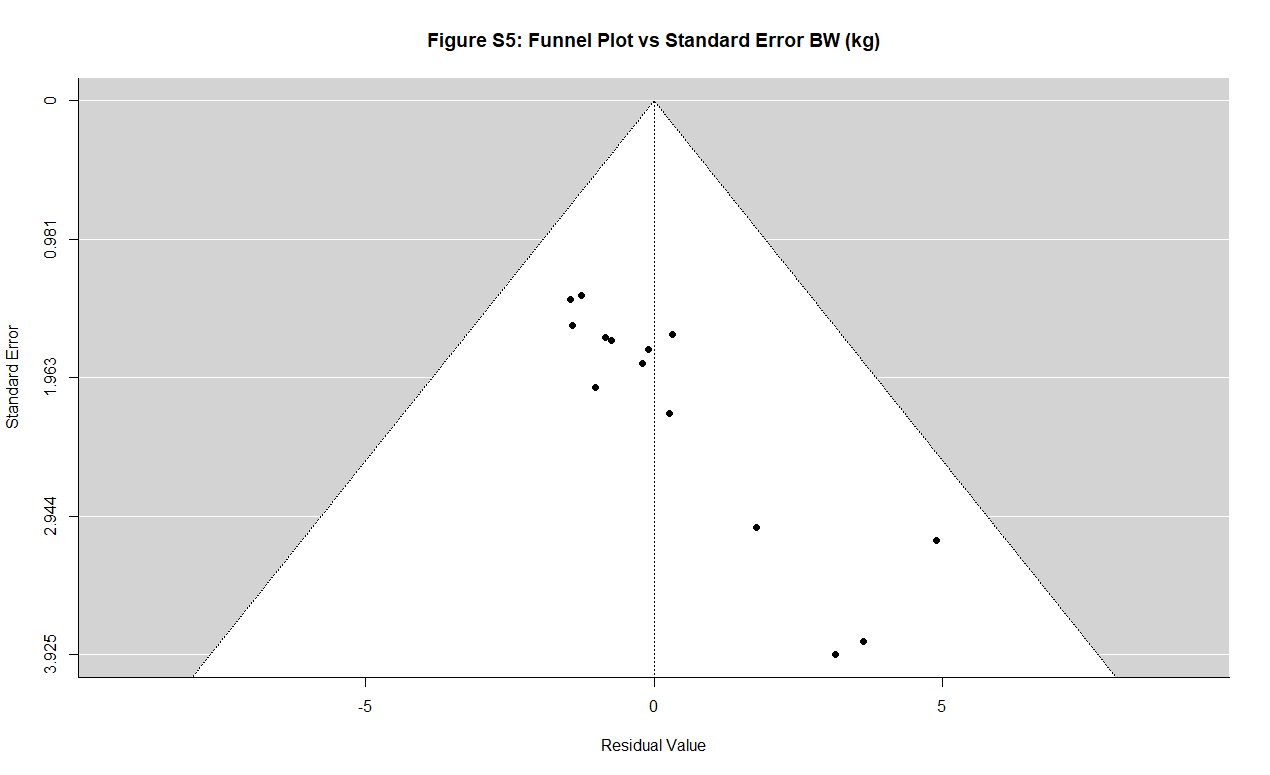


Supplementary Materials 8: Funnel plot vs standard error bodyweight (kg).


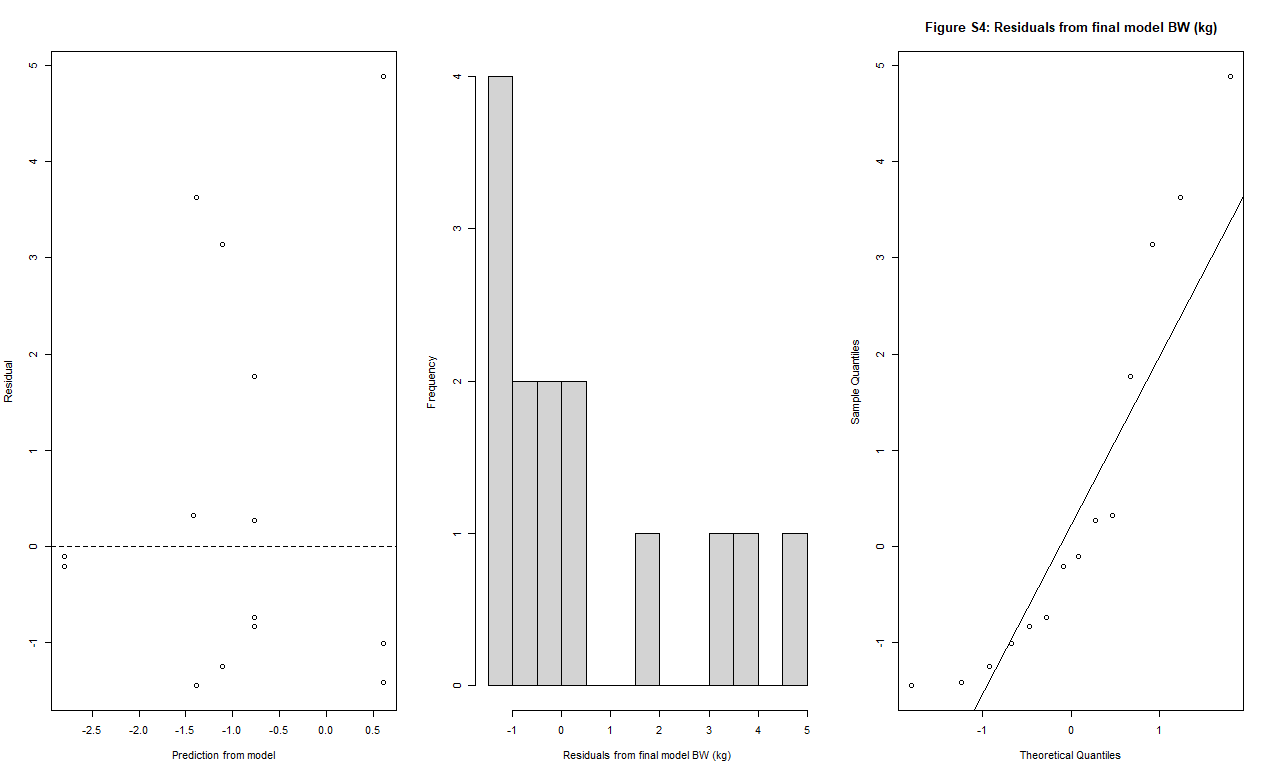


Supplementary Materials 9: Residuals for final model bodyweight (kg).


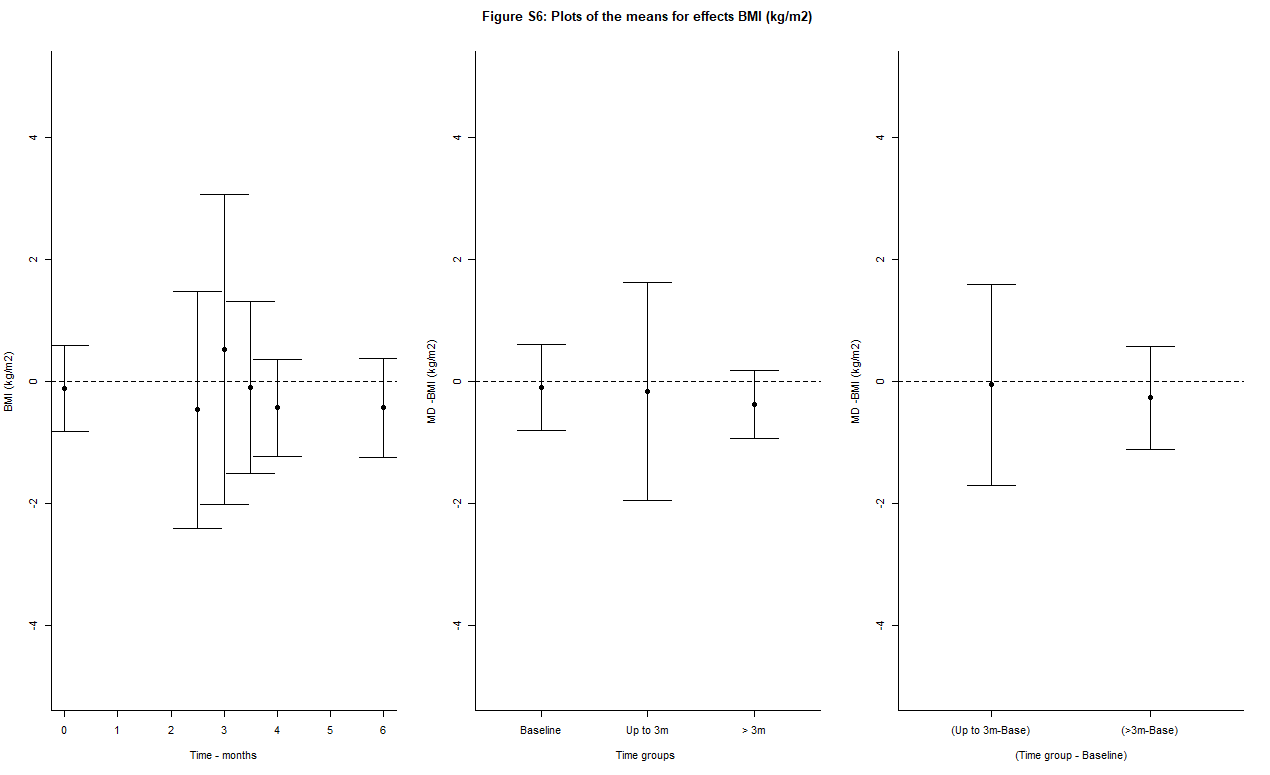


Supplementary Materials 10: Plots of means the means effect sizes BMI (kg/m^2^).


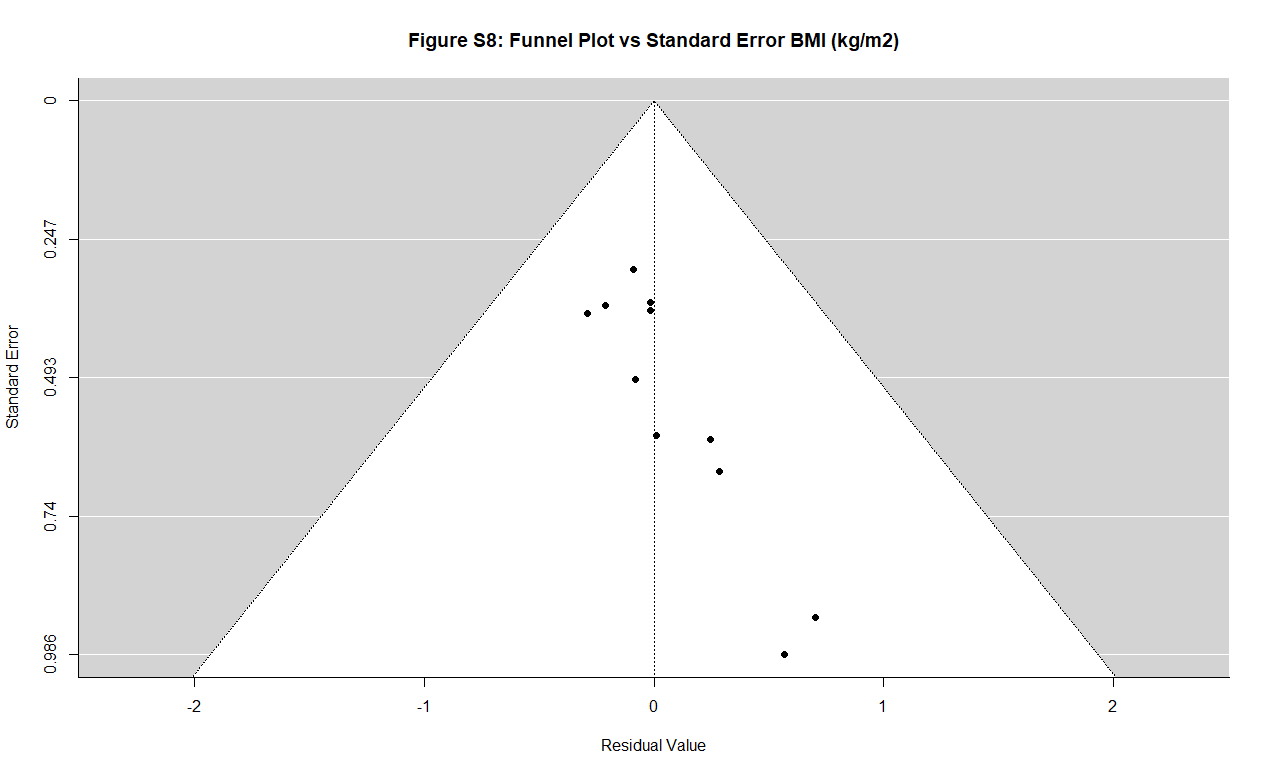


Supplementary Materials 11: Funnel plot vs standard error BMI (kg/m^2^).


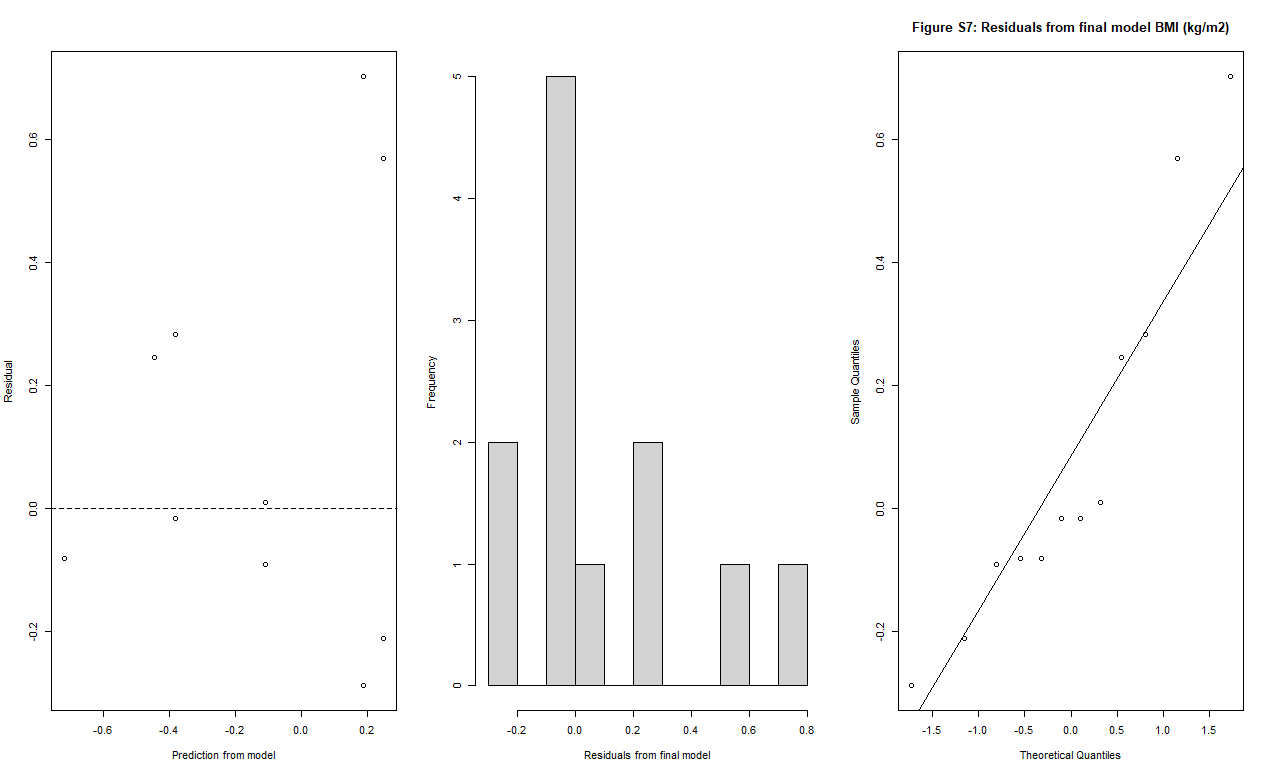


Supplementary Materials 12: Residuals for final model BMI (kg/m^2^).
